# Malaria Vaccine Development: Are Bacterial Flagellin Fusion Proteins the Bridge between Mouse and Humans?

**DOI:** 10.1155/2011/965369

**Published:** 2011-03-14

**Authors:** Daniel Y. Bargieri, Irene S. Soares, Fabio T. M. Costa, Catarina J. Braga, Luis C. S. Ferreira, Mauricio M. Rodrigues

**Affiliations:** ^1^Centro de Terapia Celular e Molecular (CTCMol), Universidade Federal de São Paulo, Escola Paulista de Medicina, Rua Mirassol 207, São Paulo 04044-010, SP, Brazil; ^2^Departamento de Microbiologia, Imunologia e Parasitologia, Universidade Federal de São Paulo, Escola Paulista de Medicina, Rua Mirassol, 207, São Paulo 04044-010, SP, Brazil; ^3^Unité de Biologie et Génétique du Paludisme, Institut Pasteur, 75724 Paris Cedex 15, France; ^4^Departamento de Análises Clínicas e Toxicológicas, Faculdade de Ciências Farmacêuticas, Avenida Prof. Lineu Prestes 580, São Paulo 05508-900, SP, Brazil; ^5^Departamento de Genética, Evolução e Bioagentes, Instituto de Biologia, Universidade Estadual de Campinas, Rua Monteiro Lobato, 255, Campinas 13083-970, SP, Brazil; ^6^Departamento de Microbiologia do Instituto de Ciências Biomédicas Universidade de São Paulo, Avenida Prof. Lineu Prestes, 1374, São Paulo 05508-900, SP, Brazil

## Abstract

In the past 25 years, the development of an effective malaria vaccine has become one of the biggest riddles in the biomedical sciences. Experimental data using animal infection models demonstrated that it is possible to induce protective immunity against different stages of malaria parasites. Nonetheless, the vast body of knowledge has generated disappointments when submitted to clinical conditions and presently a single antigen formulation has progressed to the point where it may be translated into a human vaccine. In parallel, new means to increase the protective effects of antigens in general have been pursued and depicted, such as the use of bacterial flagellins as carriers/adjuvants. Flagellins activate pathways in the innate immune system of both mice and humans. The recent report of the first Phase I clinical trial of a vaccine containing a *Salmonella* flagellin as carrier/adjuvant may fuel the use of these proteins in vaccine formulations. Herein, we review the studies on the use of recombinant flagellins as vaccine adjuvants with malarial antigens in the light of the current state of the art of malaria vaccine development. The available information indicates that bacterial flagellins should be seriously considered for malaria vaccine formulations to the development of effective human vaccines.

## 1. Whole Parasite Vaccines

### 1.1. Preerythrocytic Stages

The seminal work using radiation-attenuated sporozoites has been reproduced in multiple experimental systems, demonstrating that attenuated infective forms of *Plasmodium sp.* administered by the endovenous route can provide solid vaccination status against any symptoms of malaria [1]. However, the use of radiation-attenuated, viable live sporozoites imposes a number of restrictions because if the radiation fails, then these parasites would cause the disease, thereby nullifying any protective effects. To definitively solve this problem, a number of genetically attenuated lines of parasites have been recently generated. These genetically attenuated parasites are now being pursued as possible antigenic sources for vaccine development for humans, and Phase I and II studies are about to begin [2–5].

### 1.2. Erythrocytic Stages

Likewise, genetically attenuated blood-stage forms of rodent malaria parasites have been successfully generated in the past few years and have been proposed as an antigen source for human vaccination trials [5–7]. To our knowledge, none of these parasite lines are being tested in Phase I or II trials. Although it is technically feasible to produce large amounts of genetically attenuated sporozoites or blood-stage forms, it would be difficult to commercialise these vaccines because the parasites would have to be injected live (cryopreserved) by the endovenous route to confer protective immunity. There are also safety issues related to intravenous injections of these formulations because vaccines are not conventionally administered by this route. These two issues will certainly become the main obstacles that must be overcome for successful whole parasite vaccine development.

## 2. Recombinant Subunit Vaccines

The difficulties associated with large-scale generation of whole parasite antigens for mass vaccination led to the search for recombinant subunit vaccines based on immunodominant malarial antigens. Again, the mouse malaria model was very instructive for narrowing the search for the most promising antigens and testing different recombinant formulations, which provided the proof of principle for these subunit recombinant vaccines. Based on many years of detailed studies, some leading candidate antigens and formulations for vaccines were selected and exhaustively tested, but these tests have mainly occurred in the mouse model of infection as well as nonhuman primates.

### 2.1. Preerythrocytic Stages

Immunity generated by radiation-attenuated sporozoites is targeted to a dominant protective antigen, the circumsporozoite (CS) protein [8]. Nevertheless, other protective antigens yet to be characterized do exist [8, 9]. This protein is recognized by antibodies, CD4^+^ and CD8^+^T cells induced by immunisation with radiation-attenuated sporozoites that can eliminate the pre-erythrocytic stages of the parasite [10–14]. These results led to the development of vaccine formulations that elicit high antibody titres against sporozoites and/or increased numbers of CD4^+^ and CD8^+^ T cells specific for malaria liver stages. Although other proteins such as Liver Stage 1 or 3 and Thrombospondin-Related Anonymous Protein (TRAP) are also being explored as vaccine candidates, the CS protein can be used as a prototypical example of a pre-erythrocytic stage antigen, and the results can be extrapolated to the other surface antigens.

High antibody titres can be achieved, for example, using multiple synthetic peptides (MAPs) formulated in the presence of strong adjuvants [15–17]. Because of the success in rodent models, human safety and efficacy trials are warranted. 

The most reliable and reproducible results of vaccination against pre-erythrocytic stages in rodent malaria models were obtained by vaccination with two recombinant viral vectors. Using attenuated viruses containing the entire CS protein or its immunodominant epitopes, a solid and long-lasting protective immunity against sporozoite challenge was elicited [18–20]. Although some protocols managed to use only a single vector for vaccination [21], heterologous prime-boost vaccination using two different vectors for priming and boosting may be essential for eliciting protective immunity [22–25]. 

These exciting results obtained in the mouse model led to a number of clinical trials. Unfortunately, these trials provided only limited protective immunity in Phase II trials performed in the laboratory, and none in the field [26–28]. The reasons for such failures are difficult to explain precisely. The failures may be related to low antibody levels and/or fewer specific CD4^+^ and CD8^+^ T cells generated by these recombinant viruses in humans compared to mice. Based on the mouse model, relatively large amounts of CD4^+^ and CD8^+^ T cells are required for complete protective immunity because high numbers of these cells are already required to patrol the entire liver [13, 18, 19, 29, 30]. More powerful viral vectors and strategies of vaccination are being developed every day, and future approaches may increase the number of these protective T cells. Nevertheless, it is unclear whether it will ever be possible to reproduce in humans the levels of immunity induced by viral vectors that are found in the mouse models. 

Despite the great progress in malaria vaccine research against pre-erythrocytic stages in the past 20 years, a single type of formulation has shown promising results when tested in humans. This vaccine formulation consists of a large C-terminal fragment of the CS protein sequence fused to the Hepatitis B antigen S (conventional hepatitis B vaccine, Engerix B) and expressed as a recombinant protein in *Saccharomyces cerevisiae*. The fusion protein, named RTS, when expressed together with antigen S, naturally assembles into virus-like particles called RTS,S. The efficacy of the RTS,S formulation is dependent on the use of adjuvant systems (AS), which consist of two different formulations that include monophosphoryl lipid A (MPL, a detoxified form of LPS) and QS21 (saponin purified from *Quillaja saponaria*) in an oil-in-water emulsion (AS02) or in a liposomal suspension (AS01) [31].

In recent Phase II trials performed in naïve human volunteers challenged with *P. falciparum* sporozoites, efficacies ranging from 32% to 50% were observed. Immunological studies performed on these vaccinated individuals indicated that protection correlated with the concentration of specific antibodies and the frequency of IFN-*γ* producing cells as detected by ELISPOT [32]. A number of Phase IIb trials have been carried out, and they continue to be carried out in the field. These results are more difficult to interpret, but in trials performed in children in the endemic areas, 49.5% and 62% efficacy were reported during the 6-month period that was studied retarding the first malaria episode [33, 34]. Although partial, the protective immunity afforded by this vaccination protocol can be considered the most hopeful path for vaccine development to date. Because protective immunity was highly dependent on the adjuvants used in the formulation, these studies highlighted the importance of adjuvant development for a reliable malaria vaccine (see below). Currently, this formulation is being tested in Phase III trials in malaria-endemic areas, and the results are anxiously expected for the years 2011-12 [31].

### 2.2. Erythrocytic Stages

The erythrocytic forms of malaria grow and multiply within the host erythrocytes, and the rupture of the host cells is responsible for the clinical symptoms, including fever, of the noncomplicated forms of the disease. Nevertheless, infected erythrocytes (iEs) can also cytoadhere by means of variant surface antigens (VSA) to endothelial receptors on the microvasculature of certain organs or to the placental trophoblast, thereby disappearing from peripheral blood circulation. This phenomenon, named sequestration, is responsible for the majority of the deaths caused by malaria because it leads to the severe forms of malaria, including cerebral malaria, severe anaemia, acute respiratory distress symptoms or pregnancy-associated malaria (PAM) [35]. Because blood-stage forms of the parasite are responsible for the clinical manifestations of the disease, a vaccine against them would prevent or reduce the morbidity and mortality of malaria through the elimination or reduction of the parasitic burden (reviewed in [36, 37]). 

In the case of PAM, the scenario is complex because cytoadherence is mediated by VSA. Given that *P. falciparum *erythrocyte membrane protein-1 (PfEMP-1) is involved on parasite cytoadhesion and several studies indicate that PfEMP-1 is the major target during natural acquired immunity, this protein family is the only VSA under consideration for vaccine development based on the induction of adhesion-blocking antibodies (reviewed in [38]). Furthermore, because PAM is associated with a selection of antigenically distinct iEs that express a particular PfEMP-1 variant named VAR2CSA, a specific vaccine against this syndrome, based on this variant, should also be developed [35]. Several bodies of evidence point the VAR2CSA protein as a leading vaccine candidate against PAM and strategies based on induction of antibodies capable of inhibiting the adhesion of iEs to chondroitin sulphate A (CSA) in the placenta have recently been pursued [38, 39]. In fact, disruption of *var2csa* gene abrogates iEs CSA binding ability, plasma antibody levels against VAR2CSA are gender-specific, and naturally acquisition of high levels of VAR2CSA specific antibodies is parity-dependent and correlates to protection against PAM. However, large-scale development of a full-length VAR2CSA recombinant protein is hampered by its size (350 kDa). An alternative is the expression of all or some of its six Duffy binding-like (DBL) domains, although full-length VAR2CSA displays higher specificity and affinity to human CSA and induced antibodies abrogate adhesion of iEs to CSA [40]. Nevertheless, detailed characterization regarding VAR2CSA DBL domains interaction with CSA backbone, the resolution of their molecular structure (individually or as large fragments), and their involvement in PAM are still puzzling [38, 39].

Concerning blood-stage antigens of *Plasmodium* not directly related to PAM, in the last 20 years significant progress has been made in their molecular characterisation as well as in the identification of the immunologic mechanisms capable of eliminating these forms of the parasite (reviewed in [41–43]). Among the immunodominant antigens already characterised from blood-stage parasites, one of the main candidates for the development of a vaccine against *Plasmodium* is the Merozoite Surface Protein 1 (MSP-1) (revised in [44]). Although other proteins, such as Apical Membrane Antigen 1 (AMA-1) and Duffy-Binding Protein (DBP), are also being explored as vaccine candidates (revised in [45–47]), MSP-1 can be used as a prototypical example of a blood-stage antigen, and the results obtained from a vaccine developed around this antigen can be extrapolated to other surface antigens. 

In different species of *Plasmodium,* the structure of the C-terminal region of MSP-1 (MSP1_19_) consists of two domains. These domains contain 10–12 cysteine residues. Domain 1 or 2 contain 4–6 or 6 cysteine residues and form 2-3 or 3 disulphide bonds, respectively. Both domains are configured as epidermal growth factor-(EGF-) like domains. This type of conformation was initially suggested by the primary amino acid structure and was later confirmed by the three-dimensional analysis of recombinant proteins representing the MSP1_19_ regions of *P. cynomolgi* and *P. falciparum*. The structure of the MSP1_19_ of *P. vivax* (PvMSP1_19_) was recently elucidated through NMR, and it corroborated the MSP1_19_ structures previously described in other species [48].

During invasion of the host erythrocytes, MSP-1 is a target of proteolytic processes. Various studies have demonstrated that MSP1_42_ is found as a dimer, while MSP1_19_ remains monomeric, suggesting a secondary process that would cause the dissociation of the protein before the erythrocytes are invaded (reviewed in [44]). 

The precise biological role of MSP-1 is still unknown. However, several studies have demonstrated that antibodies specific for the MSP1_19_ of *P. falciparum *(PfMSP1_19_) can inhibit the invasion of merozoites, suggesting that this protein is involved in the process of erythrocyte invasion. In this context, attempts to knock down the coding genes of six merozoite proteins of *P. falciparum* associated with the membrane through the GPI anchor (MSP-1, MSP-2, MSP-4, MSP-5, MSP-10, RAMA, and Pf92) demonstrated that successful genetic deletion could not be obtained, with the exception of the *msp-5* gene. These observations highlight the importance of these proteins in the blood cycle of the malaria parasite [49, 50]. Finally, developmental knockout proved the importance of MSP-1 in the structure of *Plasmodium* merozoites [51]. 

Based on these positive attributes, the C-terminal region of MSP-1 (MSP1_19_ or MSP1_42_) has been heavily pursued as a vaccine candidate. Initial studies in mouse models demonstrated solid levels of protective immunity after vaccination with strong adjuvants, such as Complete Freund's Adjuvant [52, 53]. Other adjuvants did not elicit a similar effect, which clearly demonstrates the importance of the adjuvant in vaccine formulations. Although protective immunity is mostly mediated by antibodies, a recent study showed that the presence of CD4^+^ T helper epitopes improves the protective immunity induced in mouse models [54]. Subsequent studies performed in nonhuman primates duplicated these results by demonstrating a strict requirement for a strong adjuvant (e.g., Complete Freund's Adjuvant) to stimulate protective immunity against experimental infection with *P. cynomolgi* or *P. falciparum* [55, 56]. 

Based on the results obtained from the experimental models of malaria infection, a recent Phase IIb vaccine trial was performed in Africa. In this clinical trial, children were vaccinated with a formulation containing a recombinant His-tagged fusion protein encompassing the MSP1_42_ of the *P. falciparum *3D7 strain (FMP1) that was formulated with the adjuvant AS02 [57]. This vaccine formulation was shown to be safe and immunogenic, as demonstrated by detection of specific antibody titres by ELISA. Unfortunately, the trial failed, and no significant reduction in the incidence of malaria infection could be observed in children receiving the FMP1/AS02 formulation [57]. The precise reason why the vaccination failed should be investigated. It might be attributed to a polymorphism of the MSP-1 protein. This fact may be relevant to the interpretation of the results because protective immunity to the C-terminal region of *P. falciparum* MSP-1 can be strain-specific, and antibodies targeting this antigen may not show parasite inhibitory activity [56]. Furthermore, some of the MSP-1-specific antibodies are endowed with the ability to block the activity of inhibitory antibodies [44]. Although negative, these results do not completely refute the hypothesis that the C-terminal region of *P. falciparum* MSP-1 could be part of a subunit malaria vaccine in a new recombinant form or formulation. Currently, the immunogenic properties of distinct recombinant proteins are being compared in experimental animal models to select possible candidates for human trials [58].

## 3. Bacterial Flagellin Fusion Proteins: Bridging the Gap between Mice and Humans?

As thoroughly discussed above, vaccination of mice with recombinant proteins or viral vectors may confer significant protection against an infectious challenge with malaria parasites. The main problem is that some of these formulations, such as the ones containing strong adjuvants, simply cannot be used in humans. Others, such as the recombinant viruses, failed to reproduce in humans the immunogenicity observed in mice. These successive failures have led to the search for new alternatives that could eventually bridge the gap between mice and humans. 

### 3.1. Bacterial Flagellins

Flagella represent a complex locomotion device expressed by different bacterial species. They encompass at least 50 different proteins. Flagella may also behave as virulence-associated factors by helping pathogenic bacteria to bind to epithelial cells and to overcome non-immune defence mechanisms such as liquid flow in the urinary tract. Flagellin, the structural protein of the flagellar filament, is the most abundant protein of the flagellar apparatus, which can assemble by the polymerisation of thousands of flagellin monomers. The molecular masses of flagellins can vary from 28 kDa to 80 kDa. The N- and C-terminal regions of these molecules are highly conserved among different bacterial species and are folded together in parallel as a packed *α*-helix structure that is recognised by innate receptors of the immune system (see below). On the other hand, the central region shows a striking degree of sequence heterogeneity, even among flagellins from a single bacterial species. This heterogeneity has been observed in *Salmonella enterica* serovars, an immune escape mechanism reflecting the fact that most B cell epitopes are exposed to this region of the molecule [59].

Flagellin is a Pathogen-Associated Molecular Pattern (PAMP) recognised by Pattern Recognition Receptors (PRRs) in the innate immune system that triggers inflammatory responses and activates the adaptative immune system. At least two classes of PRRs, Toll-like Receptors (TLRs) and NOD-like Receptors (NLRs), respond to flagellin.

TLR5 is a transmembrane receptor with a conserved cytoplasmic signalling domain—the Toll/interleukin (IL)-1 receptor homology domain (TIR), a common signature for TLRs—that specifically recognises a conserved site in the N- and C-terminal regions of flagellin that is formed after the correct assembly of the molecule; in fact, this site is essential for the correct flagella assembly [60, 61]. The receptor is expressed by monocytes, dendritic cells (DCs), and epithelial cells that sense extracellular flagellin. When activated, TLR5 signals through the adaptor protein MyD88 to trigger NF-*κ*B activation and the subsequent expression of proinflammatory cytokines. Flagellin also induces the expression of a number of activation markers in antigen-presenting cells (APCs), such as CD80, CD86, CD40, and MHC class II, all of which augment antigen presentation to T cells [62–67]. Interestingly, flagellin is one of the two proteic agonist of TLRs.

In the case of the NLRs, flagellin that is expressed by intracellularly replicating pathogens or specifically injected by a secretory apparatus displayed by some bacterial pathogens, such as *S. enterica*, activates a multiprotein cytosolic complex, the inflammasome, which is involved in the regulation of inflammation and cell death responses in a manner dependent on ICE-protease-activating factor (Ipaf) and neuronal apoptosis inhibitory protein 5 (Naip5) [68, 69]. Activation of the inflammasome by cytosolic flagellin is related to the control of bacterial growth [70, 71] and triggers Caspase-1 activation followed by proinflammatory cell death (pyroptosis) with secretion of IL-1*β* and IL-18 and activation of inducible nitric-oxide synthase, iNOS [68, 72, 73]. A short C-terminal region of flagellin, which is distinct from the sequences required for TLR5 activation, is responsible for Naip5/Ipaf-containing inflammasome activation [69]. Nevertheless, it remains elusive whether flagellin binds directly to Naip5 or Ipaf or if it acts through an undefined upstream receptor.

Clearly, mammalian cells evolved the ability to very efficiently sense the presence of extra- and intracellular flagellins and respond with strong inflammatory signals that may ultimately enhance the induction of adaptive immune defences. Based on these natural properties, bacterial flagellins have been intensively investigated as vaccine adjuvants that can either be co-administered with purified recombinant antigens or as a carrier/adjuvant molecule genetically fused to the target antigen.

### 3.2. Bacterial Flagellins As Vaccine Adjuvants

In the late 80s, a seminal work envisaged the use of bacterial flagellins as carriers/adjuvants for vaccine antigens by cloning synthetic oligonucleotides encoding specific B or T-cell epitopes at the central hypervariable region of the *S.* Muenchen FliC*d* flagellin expressed in an attenuated *S*. Dublin vaccine strain that was delivered via mucosal or parenteral routes to mice [74]. Purified recombinant forms of flagellin genetically fused to short synthetic oligonucleotides were also successfully used as subunit vaccines by targeting either influenza virus haemagglutinin or an adhesin expressed by enterotoxigenic *Escherichia coli* strains even before the discovery that flagellin activates innate immune responses [75, 76].

More recently, the description of innate immune receptors that recognise flagellin and their capacity to activate innate and adaptive immune responses intensified the studies testing bacterial flagellins as vaccine adjuvants for the induction of both antibody and cellular immune responses. Different systems that used flagellins as carriers/adjuvants for recombinant fusion proteins or with coadministered recombinant antigens were tested in the last few years; for a detailed and updated compilation of these data, we recommend a very recently published paper [77]. 

For brevity and the purpose of our review, we selected the vaccine field that is highly advanced: the development of an anti-influenza vaccine. Two formulations of fusion proteins of flagellin and influenza antigen were generated. First, four tandem copies of the ectodomain of the conserved matrix protein M2 (M2e) of human influenza A virus were fused to the C-terminal domain of the flagellin of *Salmonella typhimurium *fljB (Type 2). A second formulation was generated by the fusion of the viral haemagglutinin globular head domain to the C-terminus of flagellin or in place of the D3 domain (VAX125). All proteins were expressed as soluble fusion proteins in the *E. coli* system. The preclinical studies testing these vaccines reported induction of strong humoral-specific immune responses against the influenza antigens after immunisation of mice and rabbits with the recombinant fusion proteins alone. In both cases, the antibody response was dependent on the physical fusion of the antigen to the flagellin. Most relevant for vaccination studies was the fact that immunisation with either construct protected mice from a lethal challenge with influenza A virus and significantly reduced weight loss and clinical symptoms compared to control animals [78, 79].

Based on these successful pre-clinical studies, the influenza vaccine candidate VAX125 underwent a complete Phase I clinical trial that tested safety, reactogenicity, immunogenicity, tolerogenicity, and escalating dose-range. The results from this trial showed that recombinant flagellin was generally well tolerated by vaccinated individuals. Importantly, 91% of the individuals who received any dose of recombinant protein developed titres of neutralising antibodies compatible with protective status against influenza infection (http://clinicaltrials.gov/NCT00921947/) [80].

### 3.3. Bacterial Flagellin As a Carrier/Adjuvant for Malaria Antigens

Based on the great capacity of flagellin to induce humoral and cellular immune responses against foreign antigens, we explored the use of this molecule for the generation of fusion proteins containing malarial antigens. We selected for our initial studies the 19-kDa C-terminal region of MSP-1 from *P. vivax. *This region of MSP-1, termed PvMSP1_19_, was selected because it is arguably the most immunogenic region of *P. vivax* [81]. In immuno-epidemiological studies performed all around the world, this molecule has been shown to be highly conserved [82] and recognised by more than 95% of individuals after their first contact with the *P. vivax* [83]. Most pertinent for vaccine development, immunisation of nonhuman primates with a recombinant protein based on PvMSP1_19_ resulted in protection against an experimental challenge [84]. 

PvMSP1_19_ was fused to the C-terminal end of the FliC flagellin from *S. typhimurium*. This fusion protein was able to bind and activate TLR5 that was expressed by *in vitro* cultured transfected cells, and it was recognised by serum from *P. vivax* malaria patients. Moreover, the fusion protein was recognised by monoclonal antibodies directed against the three-dimensional structural epitopes, indicating that it was correctly folded when expressed in the *E. coli* system [85].

We immunised mice and rabbits with the recombinant fusion protein in the absence of any other adjuvant. Immunised animals developed strong, specific, and long-lasting antibody-mediated responses. The antibody titres after the second dose were similar to the ones obtained following immunisation with a recombinant protein containing only the PvMSP1_19_ emulsified in Complete/Incomplete Freund's Adjuvant. Additionally, the antibodies raised after these immunisations recognised *P. vivax* merozoites by immunofluorescence [85]. 

In the absence of any other adjuvant, the pattern of the immune response was biased toward a type 2 response, with a high IgG1/2 ratio and limited amounts of IFN-*γ* secreted by the splenic immune cells. Nevertheless, the addition of other adjuvants to the fusion protein, such as TLR-9 agonist, modulated the immune response towards a type 1 response, with a lower IgG1/2 ratio and secretion of significantly more IFN-*γ* [85]. 

Because long-term *in vitro* culture of *P. vivax* is not feasible, and it was difficult to find a reliable biological model to test our vaccine formulation, we reproduced entirely the study using the 19-kDa C-terminal region of MSP-1 from *P. falciparum. *This region of MSP-1, termed PfMSP1_19_, was selected because it has been extensively studied as a vaccine candidate, as described above. Rabbits injected with PfMSP1_19_ fused to flagellin raised high antibody titres that dramatically inhibited the *in vitro* growth of the parasite lines 3D7, S20, and FCR3 [86]. A similar approach is now being used to generate recombinant fusion proteins containing immuno-dominant epitopes of the sporozoite stage CS protein. 

Using another approach, we inserted the CS protein CD8^+^ T-cell epitope CS_280–288_) from the murine malaria parasite *P. yoelii* into the central hypervariable (D3) domain of the *S. *Muenchen FliC flagellin [74]. The flagellin adjuvant effects were determined with two vaccine formulations: (i) attenuated *S. *Dublin strains administered orally expressing hybrid flagella composed of the flagellin-CS fusion protein and (ii) purified flagellins administered s.c. to mice either as hybrid flagellin or native flagellin mixed with synthetic CS_280–288_ peptide. Both formulations induced CS-specific CD8^+^ T-cell responses in the absence of any conventional adjuvant, as measured by ELISPOT [87]. These results suggest that *Salmonella* flagellins are promising soluble adjuvants for synthetic peptides in regard to the activation of specific cytotoxic T cell responses.

To our knowledge, these were the first reports of an induction of specific humoral and cellular immune responses in experimental immunisations with recombinant proteins based on malaria vaccine antigens without the addition of any conventional adjuvant. As mentioned above, the magnitude of an immune response induced against recombinant proteins is highly dependent on the potency of the adjuvant formulation.

## 4. Adjuvanticity Mechanism of Flagellin

The mechanisms by which flagellin acts as an adjuvant are not fully understood, but it is likely to be dependent on direct APC activation, especially DC activation, to enhance antigen presentation. Flagellin up-regulates the expression of costimulatory molecules such as B7-1, B7-2, and CD40 in murine bone marrow-derived DCs [88, 89] and B7-1, B7-2, CD83, and CCR7 in human primary blood DCs [62]. Moreover, flagellin-maturated human DCs down-regulate endocytic activity, a hallmark of DC activation, and they have enhanced T-cell stimulatory activity [62]. Another remarkable capacity of flagellin is to convert tolerogenic DCs into activated antigen-presenting cells, which is an unusual ability [90]. The effects of flagellin on DCs are at least in part dependent on TLR5 activation, as evidenced by the fact that OVA-specific CD4^+^ T cell proliferation in response to immunisation with flagellin-OVA is impaired by depletion of TLR5^+/+^CD11c^+/+^ cells or in TLR5^−/−^) mice [67]. However, TLR5^−/−^) mice still develop strong antiflagellin antibody responses after injection of flagellin; in addition, MyD88^−/−^) mice, which have impaired TLR- and NOD-dependent signalling, respond to flagellin injection with less robust antibody production [91]. In fact, recent results have shown that both TLR5 and NOD-like receptors operate in a complementary manner to support the flagellin adjuvant effects, at least in relation to the induction of humoral responses [92].

The requirement for the physical linking of flagellin to the antigen is still a matter of debate. The fusion requirement may be dependent on the amount of antigen injected. One possibility is that when flagellin is bound to the antigen, there is concomitant APC activation and antigen uptake, as opposed to coadministered soluble antigen and flagellin when an activated APC may not uptake the antigen at the same time of activation. The fact that activated DCs down-regulate endocytic activity supports this hypothesis, indicating that if activation does not happen in the presence of the antigen, uptake may be impaired, and more antigen would be required to increase the chance of concomitant antigen uptake and APC activation. In fact, when we tested equimolar amounts of flagellin and antigen that were fused or co-administered, we found that the responses are comparable in the range of 10 *μ*g; however, the fusion protein was more efficient in the induction of antigen-specific antibody responses at the levels of 1 *μ*g or 0.1 *μ*g (Bargieri DY and Rodrigues MM, unpublished data).

## 5. Conclusions

RTS,S is arguably the most advanced vaccine against *P. falciparum* malaria. Its development provided great lessons; for example, we learned that the delivery system/adjuvant is integral for the induction of any degree of immunity against malaria. Despite the on-going RTS,S Phase III clinical trials, we should not stop looking for new methods to improve the immunogenicity of recombinant antigens in humans. One possibility would be to add other recombinant vectors, such as adenoviral vectors, in combination with RTS,S to boost its potency. In this paper, we proposed an alternative approach that involved the structural modification of malarial antigens to increase their intrinsic immunogenicity. The use of recombinant fusion flagellins containing malarial epitopes may be a simple and inexpensive way to enhance protein antigenicity. The fact that recombinant flagellins have reached Phase I/II clinical trials should accelerate further studies in this direction.

## References

[1] Nussenzweig RS, Vanderberg J, Most H, Orton C (1967). Protective immunity produced by the injection of X-irradiated sporozoites of plasmodium berghei. *Nature*.

[2] Mueller AK, Labaied M, Kappe S, Matuschewski K (2005). Genetically modified Plasmodium parasites as a protective experimental malaria vaccine. *Nature*.

[3] Van Dijk MR, Douradinha B, Franke-Fayard B (2005). Genetically attenuated P36p-deficient malarial sporozouites induce protective immunity and apoptosis of infected liver cells. *Proceedings of the National Academy of Sciences of the United States of America*.

[4] VanBuskirk KM, O’Neill MT, De La Vega P (2009). Preerythrocytic, live-attenuated Plasmodium falciparum vaccine candidates by design. *Proceedings of the National Academy of Sciences of the United States of America*.

[5] Ting LM, Gissot M, Coppi A, Sinnis P, Kim K (2008). Attenuated Plasmodium yoelii lacking purine nucleoside phosphorylase confer protective immunity. *Nature Medicine*.

[6] Aly ASI, Downie MJ, Mamoun CB, Kappe SHI (2010). Subpatent infection with nucleoside transporter 1-deficient Plasmodium blood stage parasites confers sterile protection against lethal malaria in mice. *Cellular Microbiology*.

[7] Spaccapelo R, Janse CJ, Caterbi S (2010). Plasmepsin 4-deficient Plasmodium berghei are virulence attenuated and induce protective immunity against experimental malaria. *American Journal of Pathology*.

[8] Kumar KA, Sano GI, Boscardin S (2006). The circumsporozoite protein is an immunodominant protective antigen in irradiated sporozoites. *Nature*.

[9] Mauduit M, Grüner AC, Tewari R (2009). A role for immune responses against non-CS components in the cross-species protection induced by immunization with irradiated malaria sporozoites. *PLoS ONE*.

[10] Romero P, Maryanski JL, Corradin G, Nussenzweig RS, Nussenzweig V, Zavala F (1989). Cloned cytotoxic T cells recognize an epitope in the circumsporozoite protein and protect against malaria. *Nature*.

[11] Nardin EH, Herrington DA, Davis J (1989). Conserved repetitive epitope recognized by CD4 clones from a malaria-immunized volunteer. *Science*.

[12] Renia L, Marussig MS, Grillot D (1991). In vitro activity of CD4+ and CD8+ T lymphocytes from mice immunized with a synthetic malaria peptide. *Proceedings of the National Academy of Sciences of the United States of America*.

[13] Rodrigues MM, Cordey AS, Arreaza G (1991). CD8+ cytolytic T cell clones derived against the Plasmodium yoelii circumsporozoite protein protect against malaria. *International Immunology*.

[14] Malik A, Egan JE, Houghten RA, Sadoff JC, Hoffman SL (1991). Human cytotoxic T lymphocytes against the Plasmodium falciparum circumsporozoite protein. *Proceedings of the National Academy of Sciences of the United States of America*.

[15] Tam JP, Clavijo P, Lu YA, Nussenzweig V, Nussenzweig R, Zavala F (1990). Incorporation of T and B epitopes of the circumsporozoite protein in a chemically defined synthetic vaccine against malaria. *Journal of Experimental Medicine*.

[16] Nardin EH, Oliveira GA, Calvo-Calle JM, Nussenzweig RS (1995). The use of multiple antigen peptides in the analysis and induction of protective immune responses against infectious diseases. *Advances in Immunology*.

[17] Calvo-Calle JM, Oliveira GA, Watta CO, Soverow J, Parra-Lopez C, Nardin EH (2006). A linear peptide containing minimal T- and B-cell epitopes of Plasmodium falciparum circumsporozoite protein elicits protection against transgenic sporozoite challenge. *Infection and Immunity*.

[18] Bruña-Romero O, González-Aseguinolaza G, Hafalla JCR, Tsuji M, Nussenzweig RS (2001). Complete, long-lasting protection against malaria of mice primed and boosted with two distinct viral vectors expressing the same plasmodial antigen. *Proceedings of the National Academy of Sciences of the United States of America*.

[19] Reyes-Sandoval A, Berthoud T, Alder N (2010). Prime-boost immunization with adenoviral and modified vaccinia virus Ankara vectors enhances the durability and polyfunctionality of protective malaria CD8+ T-cell responses. *Infection and Immunity*.

[20] Ophorst OJ, Radošević K, Havenga MJE (2006). Immunogenicity and protection of a recombinant human adenovirus serotype 35-based malaria vaccine against Plasmodium yoelii in mice. *Infection and Immunity*.

[21] Shiratsuchi T, Rai U, Krause A, Worgall S, Tsuji M (2010). Replacing adenoviral vector HVR1 with a malaria B cell epitope improves immunogenicity and circumvents preexisting immunity to adenovirus in mice. *Journal of Clinical Investigation*.

[22] Li S, Rodrigues M, Rodriguez D (1993). Priming with recombinant influenza virus followed by administration of recombinant vaccinia virus induces CD8+ T-cell-mediated protective immunity against malaria. *Proceedings of the National Academy of Sciences of the United States of America*.

[23] Rodrigues M, Li S, Murata K (1994). Influenza and vaccinia viruses expressing malaria CD8+ T and B cell epitopes: comparison of their immunogenicity and capacity to induce protective immunity. *Journal of Immunology*.

[24] Sedegah M, Jones TR, Kaur M (1998). Boosting with recombinant vaccinia increases immunogenicity and protective efficacy of malaria DNA vaccine. *Proceedings of the National Academy of Sciences of the United States of America*.

[25] Schneider J, Gilbert SC, Blanchard TJ (1998). Enhanced immunogenicity for CD8+ T cell induction and complete protective efficacy of malaria DNA vaccination by boosting with modified vaccinia virus Ankara. *Nature Medicine*.

[26] Webster DP, Dunachie S, Vuola JM (2005). Enhanced T cell-mediated protection against malaria in human challenges by using the recombinant poxviruses FP9 and modified vaccinia virus Ankara. *Proceedings of the National Academy of Sciences of the United States of America*.

[27] Dunachie SJ, Walther M, Epstein JE (2006). A DNA prime-modified vaccinia virus Ankara boost vaccine encoding thrombospondin-related adhesion protein but not circumsporozoite protein partially protects healthy malaria-naive adults against Plasmodium falciparum sporozoite challenge. *Infection and Immunity*.

[28] Bejon P, Ogada E, Mwangi T (2007). Extended follow-up following a phase 2b randomized trial of the candidate malaria vaccines FP9 ME-TRAP and MVA ME-TRAP among children in Kenya. *PLoS ONE*.

[29] Schmidt NW, Podyminogin RL, Butler NS (2008). Memory CD8+ T cell responses exceeding a large but definable threshold provide long-term immunity to malaria. *Proceedings of the National Academy of Sciences of the United States of America*.

[30] Rodrigues M, Nussenzweig RS, Romero P, Zavala F (1992). The in vivo cytotoxic activity of CD8+ T cell clones correlates with their levels of expression of adhesion molecules. *Journal of Experimental Medicine*.

[31] Cohen J, Nussenzweig V, Nussenzweig R, Vekemans J, Leach A (2010). From the circumsporozoite protein to the RTS,S/AS candidate vaccine. *Human Vaccines*.

[32] Kester KE, Cummings JF, Ofori-Anyinam O (2009). Randomized, double-blind, phase 2a trial of falciparum malaria vaccines RTS,S/AS01B and RTS,S/AS02A in malaria-naive adults: safety, efficacy, and immunologic associates of protection. *Journal of Infectious Diseases*.

[33] Bejon P, Lusingu J, Olotu A (2008). Efficacy of RTS,S/AS01E vaccine against malaria in children 5 to 17 months of age. *New England Journal of Medicine*.

[34] Abdulla S, Oberholzer R, Juma O (2008). Safety and immunogenicity of RTS,S/AS02D malaria vaccine in infants. *New England Journal of Medicine*.

[35] Gamain B, Smith JD, Viebig NK, Gysin J, Scherf A (2007). Pregnancy-associated malaria: parasite binding, natural immunity and vaccine development. *International Journal for Parasitology*.

[36] Wipasa J, Riley EM (2007). The immunological challenges of malaria vaccine development. *Expert Opinion on Biological Therapy*.

[37] Genton B, Reed ZH (2007). Asexual blood-stage malaria vaccine development: facing the challenges. *Current Opinion in Infectious Diseases*.

[38] Hviid L (2010). The role of Plasmodium falciparum variant surface antigens in protective immunity and vaccine development. *Human Vaccines*.

[39] Dahlbäck M, Nielsen MA, Salanti A (2010). Can any lessons be learned from the ambiguous glycan binding of PfEMP1 domains?. *Trends in Parasitology*.

[40] Khunrae P, Dahlbäck M, Nielsen MA (2010). Full-length recombinant Plasmodium falciparum VAR2CSA binds specifically to CSPG and induces potent parasite adhesion-blocking antibodies. *Journal of Molecular Biology*.

[41] Reed ZH, Friede M, Kieny MP (2006). Malaria vaccine development: progress and challenges. *Current Molecular Medicine*.

[42] Yazdani SS, Mukherjee P, Chauhan VS, Chitnis CE (2006). Immune responses to asexual blood-stages of malaria parasites. *Current Molecular Medicine*.

[43] Langhorne J, Ndungu FM, Sponaas AM, Marsh K (2008). Immunity to malaria: more questions than answers. *Nature Immunology*.

[44] Kadekoppala M, Holder AA (2010). Merozoite surface proteins of the malaria parasite: the MSP1 complex and the MSP7 family. *International Journal for Parasitology*.

[45] Remarque EJ, Faber BW, Kocken CHM, Thomas AW (2008). Apical membrane antigen 1: a malaria vaccine candidate in review. *Trends in Parasitology*.

[46] Chitnis CE, Sharma A (2008). Targeting the Plasmodium vivax Duffy-binding protein. *Trends in Parasitology*.

[47] Brown A, Higgins MK (2010). Carbohydrate binding molecules in malaria pathology. *Current Opinion in Structural Biology*.

[48] Babon JJ, Morgan WD, Kelly G, Eccleston JF, Feeney J, Holder AA (2007). Structural studies on Plasmodium vivax merozoite surface protein-1. *Molecular and Biochemical Parasitology*.

[49] O’Donnell RA, Saul A, Cowman AF, Crabb BS (2000). Functional conservation of the malaria vaccine antigen MSP-1 across distantly related Plasmodium species. *Nature Medicine*.

[50] Sanders PR, Kats LM, Drew DR (2006). A set of glycosylphosphatidyl inositol-anchored membrane proteins of Plasmodium falciparum is refractory to genetic deletion. *Infection and Immunity*.

[51] Combe A, Giovannini D, Carvalho TG (2009). Clonal conditional mutagenesis in malaria parasites. *Cell Host and Microbe*.

[52] Daly TM, Long CA (1993). A recombinant 15-kilodalton carboxyl-terminal fragment of Plasmodium yoelii yoelii 17XL merozoite surface protein 1 induces a protective immune response in mice. *Infection and Immunity*.

[53] Daly TM, Long CA (1996). Influence of adjuvants on protection induced by a recombinant fusion protein against malarial infection. *Infection and Immunity*.

[54] Singh B, Cabrera-Mora M, Jiang J, Galinski M, Moreno A (2010). Genetic linkage of autologous T cell epitopes in a chimeric recombinant construct improves anti-parasite and anti-disease protective effect of a malaria vaccine candidate. *Vaccine*.

[55] Perera KLRL, Handunnetti SM, Holm I, Longacre S, Mendis K (1998). Baculovirus merozoite surface protein 1 C-terminal recombinant antigens are highly protective in a natural primate model for human plasmodium vivax malaria. *Infection and Immunity*.

[56] Lyon JA, Angov E, Fay MP (2008). Protection induced by plasmodium falciparum MSP1 is strain-specific, antigen and adjuvant dependent, and correlates with antibody responses. *PLoS ONE*.

[57] Ogutu BR, Apollo OJ, McKinney D (2009). Blood stage malaria vaccine eliciting high antigen-specific antibody concentrations confers no protection to young children in Western Kenya. *PLoS ONE*.

[58] Reed ZH, Kieny MP, Engers H (2009). Comparison of immunogenicity of five MSP1-based malaria vaccine candidate antigens in rabbits. *Vaccine*.

[59] Ramos HC, Rumbo M, Sirard JC (2004). Bacterial flagellins: mediators of pathogenicity and host immune responses in mucosa. *Trends in Microbiology*.

[60] Hayashi F, Smith KD, Ozinsky A (2001). The innate immune response to bacterial flagellin is mediated by Toll-like receptor 5. *Nature*.

[61] Smith KD, Andersen-Nissen E, Hayashi F (2003). Toll-like receptor 5 recognizes a conserved site on flagellin required for protofilament formation and bacterial motility. *Nature Immunology*.

[62] Means TK, Hayashi F, Smith KD, Aderem A, Luster AD (2003). The toll-like receptor 5 stimulus bacterial flagellin induces maturation and chemokine production in human dendritic cells. *Journal of Immunology*.

[63] Arimilli S, Johnson JB, Clark KM (2008). Engineered expression of the TLR5 ligand flagellin enhances paramyxovirus activation of human dendritic cell function. *Journal of Virology*.

[64] Merlo A, Calcaterra C, Ménard S, Balsari A (2007). Cross-talk between Toll-like receptors 5 and 9 on activation of human immune responses. *Journal of Leukocyte Biology*.

[65] Agrawal S, Agrawal A, Doughty B (2003). Cutting Edge: different Toll-like receptor agonists instruct dendritic cells to induce distinct Th responses via differential modulation of extracellular signal-regulated kinase-mitogen-activated protein kinase and c-Fos. *Journal of Immunology*.

[66] Didierlaurent A, Ferrero I, Otten LA (2004). Flagellin promotes myeloid differentiation factor 88-dependent development of Th2-type response. *Journal of Immunology*.

[67] Bates JT, Uematsu S, Akira S, Mizel SB (2009). Direct stimulation of tlr5+/+ CD11c+ cells is necessary for the adjuvant activity of flagellin. *Journal of Immunology*.

[68] Miao EA, Alpuche-Aranda CM, Dors M (2006). Cytoplasmic flagellin activates caspase-1 and secretion of interleukin 1*β* via Ipaf. *Nature Immunology*.

[69] Lightfield KL, Persson J, Brubaker SW (2008). Critical function for Naip5 in inflammasome activation by a conserved carboxy-terminal domain of flagellin. *Nature Immunology*.

[70] Zamboni DS, Kobayashi KS, Kohlsdorf T (2006). The Birc1e cytosolic pattern-recognition receptor contributes to the detection and control of Legionella pneumophila infection. *Nature Immunology*.

[71] Vinzing M, Eitel J, Lippmann J (2008). NAIP and Ipaf control Legionella pneumophila replication in human cells. *Journal of Immunology*.

[72] Buzzo CL, Campopiano JC, Massis LM (2010). A novel pathway for inducible nitric-oxide synthase activation through inflammasomes. *Journal of Biological Chemistry*.

[73] Bortoluci KR, Medzhitov R (2010). Control of infection by pyroptosis and autophagy: role of TLR and NLR. *Cellular and Molecular Life Sciences*.

[74] Newton SMC, Jacob CO, Stocker BAD (1989). Immune response to cholera toxin epitope inserted in Salmonella flagellin. *Science*.

[75] McEwen J, Levi R, Horwitz RJ, Arnon R (1992). Synthetic recombinant vaccine expressing influenza haemagluttinin epitope in Salmonella flagellin leads to partial protection in mice. *Vaccine*.

[76] Luna MG, Martins MM, Newton SMC, Costa SOP, Almeida DF, Ferreira LCS (1997). Cloning and expression of colonization factor antigen I (CFA/I) epitopes of enterotogenic *Escherichia coli* (ETEC) in Salmonella flagellin. *Research in Microbiology*.

[77] Mizel SB, Bates JT (2010). Flagellin as an adjuvant: cellular mechanisms and potential. *Journal of Immunology*.

[78] Huleatt JW, Nakaar V, Desai P (2008). Potent immunogenicity and efficacy of a universal influenza vaccine candidate comprising a recombinant fusion protein linking influenza M2e to the TLR5 ligand flagellin. *Vaccine*.

[79] Song L, Nakaar V, Kavita U (2008). Efficacious recombinant influenza vaccines produced by high yield bacterial expression: a solution to global pandemic and seasonal needs. *PLoS ONE*.

[80] Treanor JJ, Taylor DN, Tussey L (2010). Safety and immunogenicity of a recombinant hemagglutinin influenza-flagellin fusion vaccine (VAX125) in healthy young adults. *Vaccine*.

[81] Barbedo MB, Ricci R, Jimenez MCS (2007). Comparative recognition by human IgG antibodies of recombinant proteins representing three asexual erythrocytic stage vaccine candidates of Plasmodium vivax. *Memorias do Instituto Oswaldo Cruz*.

[82] Soares IS, Barnwell JW, Ferreira MU (1999). A Plasmodium vivax vaccine candidate displays limited allele polymorphism, which does not restrict recognition by antibodies. *Molecular Medicine*.

[83] Rodrigues MHC, Cunha MG, Machado RLD, Ferreira OC, Rodrigues MM, Soares IS (2003). Serological detection of Plasmodium vivax malaria using recombinant proteins corresponding to the 19-kDa C-terminal region of the merozoite surface protein-1. *Malaria Journal*.

[84] Dutta S, Kaushal DC, Ware LA (2005). Merozoite surface protein 1 of Plasmodium vivax induces a protective response against Plasmodium cynomolgi challenge in rhesus monkeys. *Infection and Immunity*.

[85] Bargieri DY, Rosa DS, Braga CJM (2008). New malaria vaccine candidates based on the Plasmodium vivax Merozoite Surface Protein-1 and the TLR-5 agonist Salmonella Typhimurium FliC flagellin. *Vaccine*.

[86] Bargieri DY, Leite JA, Lopes SCP (2010). Immunogenic properties of a recombinant fusion protein containing the C-terminal 19 kDa of Plasmodium falciparum merozoite surface protein-1 and the innate immunity agonist FliC flagellin of Salmonella Typhimurium. *Vaccine*.

[87] Braga CJM, Massis LM, Sbrogio-Almeida ME (2010). CD8+ T cell adjuvant effects of Salmonella FliCd flagellin in live vaccine vectors or as purified protein. *Vaccine*.

[88] Pino O, Martin M, Michalek SM (2005). Cellular mechanisms of the adjuvant activity of the flagellin component FljB of Salmonella enterica serovar typhimurium to potentiate mucosal and systemic responses. *Infection and Immunity*.

[89] Datta SK, Redecke V, Prilliman KR (2003). A subset of toll-like receptor ligands induces cross-presentation by bone marrow-derived dendritic cells. *Journal of Immunology*.

[90] Vicente-Suarez I, Brayer J, Villagra A, Cheng F, Sotomayor EM (2009). TLR5 ligation by flagellin converts tolerogenic dendritic cells into activating antigen-presenting cells that preferentially induce T-helper 1 responses. *Immunology Letters*.

[91] Sanders CJ, Franchi L, Yarovinsky F (2009). Induction of adaptive immunity by flagellin does not require robust activation of innate immunity. *European Journal of Immunology*.

[92] Vijay-Kumar M, Carvalho FA, Aitken JD, Fifadara NH, Gewirtz AT (2010). TLR5 or NLRC4 is necessary and sufficient for promotion of humoral immunity by flagellin. *European Journal of Immunology*.

